# New York Academy of Medicine

**Published:** 1868-04

**Authors:** Alfred C. Post

**Affiliations:** President, in the Chair


### NEW YORK ACADEMY OF MEDICINE.

STATED MEETING DEC. 4th, 1867,

DR. ALFRED C. POST, President, in the Chair.

### ON BLOOD-LETTING.

Dr. Post, by previous arrangement, opened the discussion upon the subject of blood-letting. After attending to the antiquity of the practice—it having been in vogue among the Egyptians, Assyrians, and other nations, and resorted to by Polydorus soon after the seige of Troy, endorsed by Hippocrates, and its rank as a remedial agent all through the period of Roman civilization,—he remarked as follows:

Notwithstanding its loss of reputation, partly caused by the abuse of its power, he contended that venesection was a remedy of great value, and in many cases it was indispensable; in other words, that it shortened the period of disease, and increased the prospect of cure.

Its effect upon the circulation was well marked, as evidenced by a decrease in the frequency of the pulse, a lowering of the animal heat, and a diminution in the volume of the artery. To use a conventional phrase, "the pulse rises," it becomes fuller, and the patient very often feels immediate relief. The effect of opening a vein is beautifully illustrated in peritoneal inflammation, for here the pulse being "*small*," an increase in force is more readily detected.

In acute inflammation, and in active determination of blood to important organs, the blood-letting plan of treatment was of substantial value. It relieved local pain. Among the other effects the Doctor alluded to free and copious perspiration, a tendency to syncope, nausea, emesis, and sometimes convulsions.

It may be judiciously employed from infancy to old age, although in the first mentioned stage of life, its application, perhaps for no very cogent reasons, was limited to the use of leeches. In advanced age great caution is required, but for the benefit of the over-timid, he cited two recorded cases. One that of a man aged 87, bled twice in one day; another aged 80, bled nine times for pneumonia. These in proof that even under this adverse condition, it was not so destructive to life after all.

The case of the late Dr. Cheesman, (who died in 1862, aged 75 years,) as related by Dr. Alonzo Clark, was also to be remembered in this connection, since he was evidently benefited by a copious bleeding.

Dr. Post then related several cases to fortify his position, one or two of which are here given.

A woman *æt.* 40, small and slender, with a pulse of inconsiderable force, but not remarkably compressible, being seized with a severe cephalalgia referable to the frontal region, sent for him. He administered the usual remedies, revulsive, cathartics, etc., but without effect. The patient then suggested bleeding, which Dr. P. had not before entertained, owing to its apparent contraindication. Her solicitation was backed by the assurance that previous bleedings in similar attacks had always afforded relief. The loss of something over a pint of blood from her arm verified her statement. He was subsequently called at long intervals, and bled the patient each time with the same result. After several years also, he was summoned at night to her bedside; she was in state of stupor, had a wild look, was unable to speak, but significantly pointed to her elbow. A full bleeding brought relief, and since then she has enjoyed comparative immunity from attack.

Case 2. A young clergyman, in the course of a platform speech, reeled and fell. Having a flushed face, full pulse, and great confusion of mind, he was bled, with marked and complete relief.

Dr. Post, in concluding his remarks, cited the case of strangulated hernia at the New York Hospital, upon which he had called the usual consultation of his colleagues, preliminary to an operation. In the interim, however, he directed the hard, tender, and tense tumor to be covered with leeches, when spontaneous reduction took place. He also referred to the benefits of the local abstraction of blood by leeches in the case of pneumonia in the very young subject, and in pneumonia generally, when no depressing influence is at work.

Dr. Anderson referred to the change in professional opinion regarding this matter, which would be more obvious by contrasting a period 25 years ago with the present time. He remembered having read the work of Dr. Armstrong on fevers, in which venesection was much praised, and it had occurred to him that following up the bleeding, with anodynes, would be eminently philosophical. While of this way of thinking, a gentleman, "a full, hearty liver," sent for him. The patient had great respiratory oppression, but no pneumonia; bleeding at once relieved him. In the case of Dr. Cheesman it would be proper to note, that more blood was lost than was originally intended, since the slipping of the bandage allowed the bed to become quite well ensanguined before the discovery was made.

Remarks were made by Drs. Woodhull, Foster, and Garish.

Dr. Van Buren said, at the beginning of his professional career it was thought impossible to initiate the treatment of pleurisy without a resort to blood-letting. In such cases the respiration did certainly become quite free. He also alluded to the benefit accruing from venesection in two cases of puerperal convulsions, before the days of chloroform.

Dr. Wooster gave in his testimony regarding the application of leeches between the shoulder blades,—especially in the case of young children suffering from pneumonia of a grave type. He was in every sense well pleased with the result.

Dr. Sayre was cognizant of a case where leeches were applied between the shoulders, and the child died in consequence. This had made a strong impression on him. Oiled silk jackets, poultices, etc., he regarded as much safer, and more serviceable.

Cases requiring prompt bleeding he regarded as quite rare. He did not wish to be understood as saying they never occurred, since he had himself carried a lancet in his vest pocket for 15 years, and had used it three times.

The change in our modes of treatment were due, he remarked, not to popular prejudice, but to the increased information of medical men. The starvation and the lancet plan had gone out of date; all were beginning to believe more in good food and good air.

Dr. Woodhull would hesitate to bleed, except in cases of sudden apoplexy.

Dr. Richards, after a few remarks by Dr. Van Buren concerning a change of type in disease, asked whether it might not be a change of opinion, and for the purpose of giving the junior members of the Academy an opportunity of expressing their views, he moved that the discussion be resumed at the first opportunity.

The motion prevailed, and the Academy adjourned.

*Stated Meeting, Feb. 5th, 1868.* Dr. Alfred C. Post President, in the Chair. After the usual routine, business was transacted.

Dr. D. B. St. John Roosa read a paper upon *Congenital Deaf Mutism.* A short discussion ensued, when the President declared the continuation of the discussion on blood-letting to be in order.

BLOOD-LETTING.

Dr. O'Sullivan made the following remarks on the therapeutic effects of blood-letting:

Mr. President: The discussion on blood-letting, and the information elicited on that subject at a former meeting of the Academy, has been one of the most interesting and instructive to me as one of the Junior Fellows. The earliest lessons which I have learned in the study of medicine, have been intimately connected with the ideas prevailing at the time as regards blood-letting, and which governed the practice some years since.

When I commenced practice, I determined with all the zeal of a young practitioner, to test the merits of blood-letting thoroughly. In the neighborhood where I have resided for the past 11 years, there are several large founderies and machine shops, where hundreds of stalwart men are constantly employed. Here was a wide field for investigation, and so determined was I to give the subject a fair trial, that for several years I never went without a lancet in my pocket. I, however, began to perceive very soon that among this class of patients, who were apparently well able to stand depletion, I had to alter my views materially, and finally to abandon almost entirely the use of the lancet, especially in diseases affecting the respiratory organs. Now, sir, whether this is owing to the fact that this class of patients reside in ill-ventilated and crowded apartments, or that in their daily avocations they are constantly breathing a vitiated and poisoned atmosphere, or else to climatic reasons, I am not at present prepared to say. The fact however remains, that a certain degree of enervation exists among this class of patients, and that we must be very careful how we deplete them. In acute diseases of children I have never pursued the practice. I can say with a clear conscience, that I have never even applied a single leech, but on the contrary, endeavored to sustain my little patients from the beginning, in every possible way, locally; the oil silk jacket and poultices sufficiently fulfil the indications for depletion.

In this connection I will briefly mention the history of an interesting case which occurred in my practice some eight years ago, and which I think strongly sustains my views on the subject.

In January, 1860, I received a telegram urgently calling me to Baltimore, to see a relative, who was a student at the theological seminary in that city. I left immediately by the Night Express train, arriving at five A.M. I at once proceeded to the seminary, and found the patient in a very debilitated condition, in the second stage of pneumonia; and, as I was informed by the nurse, and later by the medical attendant, that he had had no sleep for three days and nights. It was evident to me at a single glance, that if sleep was not speedily procured, delirium must ensue, and with necessarily a fatal result. I was further informed by both the physician and nurse, that at the onset of the disease the patient had been bled very freely (a large basin full of blood being taken from him) and that considerable debility had followed the venesection, and from which he had but imperfectly rallied when I saw him. The state of affairs can be readily imagined, when it is known that the patient was a mere anæmic youth of 19 years of age; of course, I need hardly say that the antiphlogistic regimen was also pursued. On auscultation, I found he had a very extensive pneumonia, involving both the lower and middle lobes of the lung, there had been applied extensive counter-irritation on the obsolete theory of its furthering resolution, a mode of treatment which the best clinical observers of the day have so fully refuted. In relating particulars, the attending physician expressed his surprise, that notwithstanding he had administered large doses of opium in a concentrated form, no sleep had been procured, and that in his opinion the case was very serious, and an immediate fatal result was highly probable. He asked my views of the case, and what I would suggest in the present emergency. I said I very much regretted the treatment he had pursued in the earlier stages of the disease, that in so young and weak a patient, instead of depletion, sustaining treatment of a decided character should have been pursued from the very beginning, that had such a course been adopted, the solidification would not be so extensive, nor the debility be so extreme. I suggested at once the most heroic sustaining treatment; beef-tea, tonics, stimulants, etc. Being then unacquainted with any of the profession in Baltimore, I telegraphed to a distinguished Fellow of this Academy, who kindly sent me the names of some prominent physicians in Baltimore, with one of whom an immediate consultation was had, and who fully endorsed my views of the treatment of the case; and on the first night on a few grains of Dover's powder the patient enjoyed three hours of most refreshing sleep, resting as placidly as an infant. I, with the nurse, watched him the whole night. Now for the sequel: the pneumonia was cured, but the patient was lost, as he died of phthisis some six months later. The deduction I have drawn is, that the immediate and predisposing cause of the fatal result in this case, is attributable to excessive depletion on the one hand, and defective nutrition on the other. I would mention further, that the young man inherited no predisposition to the disease of which he died—his relatives on both sides were not tainted with the disease, many of them living to an extreme old age. During the collegiate course of the patient in New York city, he was under my constant supervision, and was never to my knowledge sick for a single day.

No further remarks having been offered, the meeting adjourned.—*Phila. Medical & Surgical Reporter.*

